# Ionising Radiation Promotes Invasive Potential of Breast Cancer Cells: The Role of Exosomes in the Process

**DOI:** 10.3390/ijms222111570

**Published:** 2021-10-26

**Authors:** Raheem AL-Abedi, Seda Tuncay Cagatay, Ammar Mayah, Susan A. Brooks, Munira Kadhim

**Affiliations:** Department of Biological and Medical Sciences, Oxford Brookes University, Oxford OX3 0BP, UK; abediraheem19@gmail.com (R.A.-A.); stuncay-cagatay@brookes.ac.uk (S.T.C.); mayahahj@gmail.com (A.M.); sbrooks@brookes.ac.uk (S.A.B.)

**Keywords:** exosomes, breast cancer, RIBE, invasiveness, EMT, glycosylation

## Abstract

Along with the cells that are exposed to radiation, non-irradiated cells can unveil radiation effects as a result of intercellular communication, which are collectively defined as radiation induced bystander effects (RIBE). Exosome-mediated signalling is one of the core mechanisms responsible for multidirectional communication of tumor cells and their associated microenvironment, which may result in enhancement of malignant tumor phenotypes. Recent studies show that exosomes and exosome-mediated signalling also play a dynamic role in RIBE in cancer cell lines, many of which focused on altered exosome cargo or their effects on DNA damage. However, there is a lack of knowledge regarding how these changes in exosome cargo are reflected in other functional characteristics of cancer cells from the aspects of invasiveness and metastasis. Therefore, in the current study, we aimed to investigate exosome-mediated bystander effects of 2 Gy X-ray therapeutic dose of ionizing radiation on the invasive potential of MCF-7 breast cancer cells in vitro via assessing Matrigel invasion potential, epithelial mesenchymal transition (EMT) characteristics and the extent of glycosylation, as well as underlying plausible molecular mechanisms. The findings show that exosomes derived from irradiated MCF-7 cells enhance invasiveness of bystander MCF-7 cells, possibly through altered miRNA and protein content carried in exosomes.

## 1. Introduction

Exosomes are membrane-bound, nanosized vesicles of endosomal origin that can be released from almost all types of cells [1,2]. Exosomes participate in cell-cell communication by carrying cytosolic cargo of donor cells including proteins, nucleic acids, lipids, metabolites which can be delivered to the recipient/target cells [3,4,5,6]. They have been shown to play a dynamic role in many aspects of carcinogenesis and cancer progression including enhancing invasiveness, angiogenesis and chemoresistance of other tumour cells via transport of oncogenic elements [7,8,9]. It is established that exosomes derived from healthy cells and cancer cells are distinct in terms of the rate of exosomal release [10,11]. It has also been well documented that the content of exosomes derived from tumours, mainly miRNA, protein and lipid profile, differ between normal and cancer cells. Exosomes derived from malignant mesothelioma cell lines established from clinical tumors facilitate the migration of fibroblast/endothelial cells due to unique oncogenic signature present in those exosomes, as profiled by biophysical and proteomic characterisation [12]. In another clinical study investigating patients with metastatic melanoma, exosomal miRNAs were found to be differentially expressed in different patient cohorts [13]. Moreover, exosomes derived from urine samples from patients with prostate cancer have been shown to cargo a distinctive lipid profile compared those from healthy individuals [14]. Breast cancer exosomes derived from MCF-7 cells have been shown to cargo specific lipid molecules [15].

Recent studies have also shown that exosomes can modify the tumour microenvironment to enhance tumour cell invasion, metastasis and EMT [16]. EMT is the process by which epithelial cells are transformed into cells with mesenchymal phenotypes through modulation of pathways which include the SNAIL family of zinc-finger transcription factors, SNAIL/SLUG, ZEB family, TWIST1/TWIST2 family transcription factors as well as noncoding RNA inducers, mainly miRNAs [17]. This complex interactome can communicate through multiple signals received from the proximal microenvironment and reprogramme gene expression [18]. EMT is a crucial step in cancer cell migration and invasion [19,20]. TGF-β acts as a multifunctional and EMT promoting cytokine in many cancer cells [21,22,23]. It has been well established that TGF-β can instigate cancer progression by inducing EMT either directly through activation of EMT transcription factors [24] or through other pathways such as p38 MAPK [25] and PI3K/AKT/mTOR [26]. The complex pathway of intracellular signalling through TGF-β involves the glycosylation of cell surface TGF-β-binding proteins [27,28] which has critical importance for the cellular responses induced by TGF-β, including the EMT.

Intriguingly, another hallmark of cancer that is associated with invasion and metastasis in many human malignancies is aberrant glycosylation, which can be detected by lectin histochemistry [29]. In one of the two main types of glycosylation, *O*-linked glycosylation, an *N*-acetylgalactosamine (GalNAc) residue is added to the hydroxyl group of Serine or Threonine residue on the polypeptide [30]. Several studies have linked an increased display of GalNAc to poor prognosis and metastatic competence that can be detected by the lectin *Helix pomatia* agglutinin (HPA) binding in a wide variety of cancers including those of breast [31,32,33,34], thyroid [35], oesophagus [36], stomach [37], colorectum [38], lung [39] and prostate [40].

Radiation therapy is one of the most common approaches to kill cancer cells and shrink tumours in the course of cancer treatment [41]. Concordantly, studies regarding the non-targeted effects (NTEs) of ionising radiation have been intensified over the recent years. Ionising radiation (IR) induces complex, global cellular responses in both irradiated and non-irradiated ‘bystander’ cells which receive molecular signals produced by irradiated cells [42]. Bystander effects are a type of NTE observed in non-irradiated cells that were either in contact with, or received soluble signals from, irradiated cells [43]. Exosome signalling is one of the mechanisms through which bystander effects can be transmitted. IR can cause increased exosome release that act as a messenger of bystander effects in both normal [44] and cancer cells [45]. Evidence for the importance of exosomes and exosomal content, particularly miRNAs, for transmitting bystander effects have been increased over the recent years. For example, it has been shown that miR-7-5p can induce autophagy in non-targeted BEP2D human bronchial epithelial cells [46]. Studies carried out with the human normal embryonic lung fibroblast cell line MRC-5 demonstrated that miR-21 can act as a bystander signalling molecule by shuttling through irradiated and non-irradiated cells via exosomal transport [47,48]. In relation to cancer, it has been shown that radiation can increase the number of exosomes released by glioblastoma cells and exosomes derived from irradiated cells exacerbate the migration of recipient cells, possibly through increasing cell migration signalling molecules, as shown by molecular profiling [49]. It has also been demonstrated that exosomes in conditioned media of MCF-7 cells can induce bystander effects in non-irradiated MCF-7 cells in terms of DNA damage [45]. Moreover, exosomes derived from the head and neck squamous cancer cell line BHY following ionizing radiation promotes growth and survival of non-irradiated cells [50].

Although mounting evidence suggests that exosomes are actively involved in modulating cancer cell phenotypes, there is a lack of knowledge regarding cancer cell-derived exosomes in the context of bystander effects of IR. Therefore, in this study we aimed to investigate the bystander effects transduced by exosomes in MCF-7 breast cancer epithelial cells following 2 Gy therapeutic dose of X-ray irradiation regarding their invasive potential by focusing on functional invasive characteristics, HPA binding, EMT marker analysis and gene expression analysis.

## 2. Results

### 2.1. Investigation of Invasive Potential of MCF-7 Cells Following Conditioned Media Transfer

**Figure 1:**
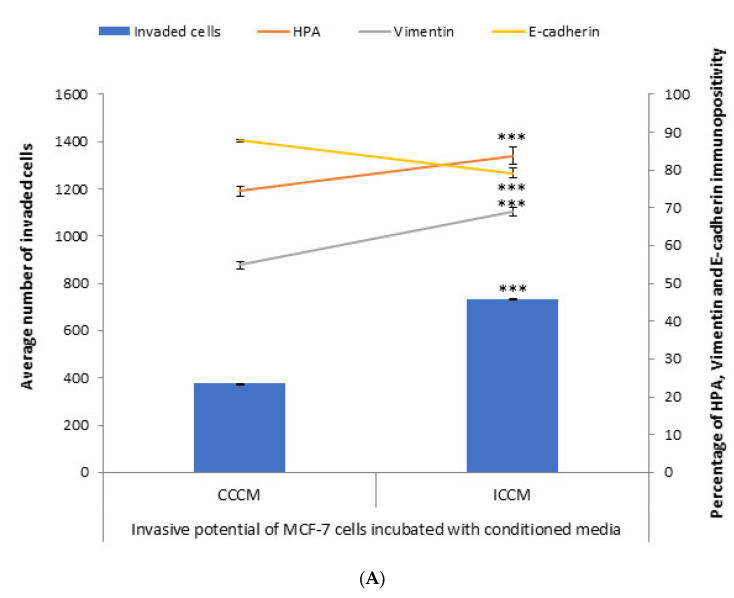

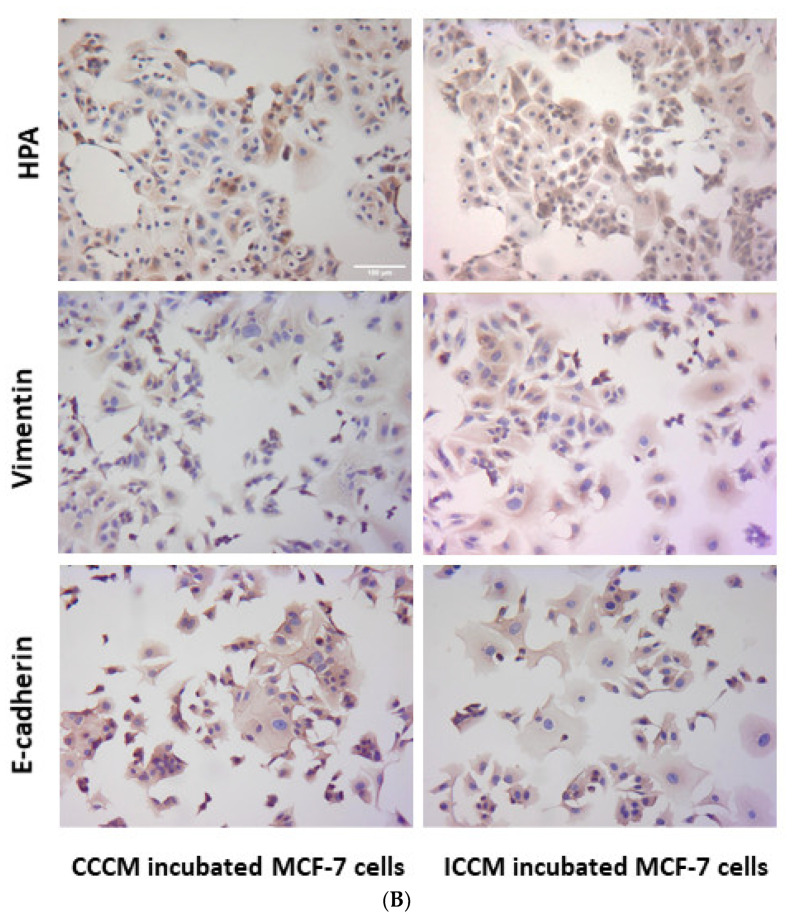
Invasive potential of MCF-7 cells shown by Matrigel invasion assay, glycosylation and EMT characteristics following 24 h CCCM or ICCM transfer. (**A**) The data presented as a mean of a total number of invaded cells, and the percentage of HPA labelling and EMT markers (vimentin and E-cadherin) positive cells. The error bars represent the SEM of invaded cells and the percentage of HPA and EMT markers of 3 independent experiments (*** *p* ≤ 0.0001); (**B**) Representative images for HPA labelling and EMT markers (vimentin and E-cadherin) assays in CCCM or ICCM incubated MCF-7 cells. Scale bar: 100 μm.

In order to test the bystander effects of ionizing radiation from the aspect of invasiveness and metastasis, irradiated cells conditioned media (ICCM) from 2Gy X-ray irradiated and control cells conditioned media (CCCM) from sham-irradiated MCF-7 cells was introduced onto unirradiated MCF-7 cells. We assessed the invasive potential via evaluation of the number of invaded cells through Matrigel, EMT markers immunopositivity and glycosylation of MCF-7 cells. Following 24 h incubation, cells were analysed for the number of invaded cells, HPA binding, and EMT markers immunopositivity. As shown in Figure 1, the number of cells that had invaded through the transmembrane system was significantly higher for MCF-7 cells incubated with ICCM compared to those incubated with CCCM, in parallel with a significant increase in the percentage of vimentin-positive cells and a significant decrease in the percentage of E-cadherin-positive cells. Concurrent with these findings, the percentage of HPA-positive cells was significantly higher for MCF-7 cells incubated with ICCM compared to MCF-7 cells incubated with CCCM.

### 2.2. Investigation of Invasive Potential of MCF-7 Cells Following Exosome Transfer

#### 2.2.1. *Characterisation of Exosomes by qNano*

**Figure 2:**
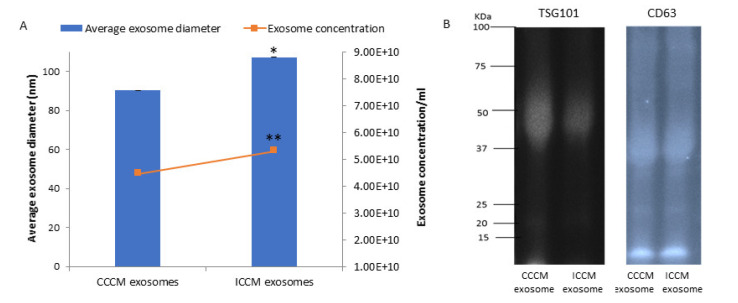
Characterisation of exosomes derived from CCCM and ICCM via concentration, size and presence of exosomal markers. (**A**) Concentration and average size of exosomes obtained from CCCM and ICCM exosome suspensions. The data were presented as a mean ± SEM of 3 independent experiments (* *p* ≤ 0.05, ** *p* ≤ 0.001. (**B**) Western blot analysis of exosomes for TSG101 (44 kDa) and CD63 (26 kDa) exosomal markers. TSG101 blot; Lane 1: CCCM exosome protein, Lane 2: ICCM exosome protein. CD63 blot; Lane 1: CCCM exosome protein, Lane 2: ICCM exosome protein.

Exosomes were extracted from CCCM or ICCM using ultracentrifugation. Exosome size and concentration of samples were analysed via qNano analysis. Results showed that exosome concentration was significantly higher in ICCM (5.30 × 10^10^/mL) compared to CCMM (4.47 × 10^10^/mL). Similarly, average exosome size was increased in ICCM showing that ionising radiation altered both concentration and size distribution of exosomes released into the medium by post-IR MCF-7 cells as shown in Figure 2A.

#### 2.2.2. *Characterisation of Exosomes by Western Blot*

In order to further confirm the presence of exosomes, CCCM and ICCM exosome samples were investigated via Western blot for two common exosome markers, TSG101 and CD63. Figure 2B confirms the presence of both markers in the exosome samples derived from CCCM or ICCM exosomes.

#### 2.2.3. *Invasive Potential of MCF-7 Cells*

**Figure 3:**
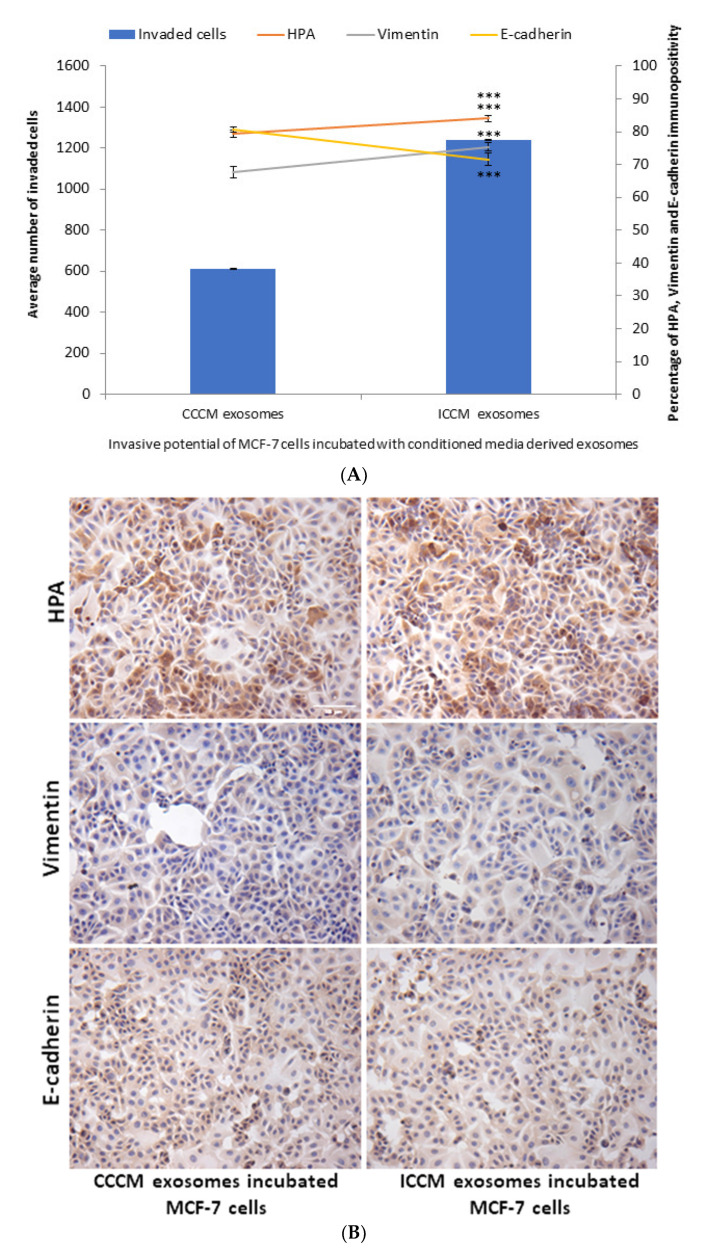
Invasive potential of MCF-7 cells shown by Matrigel invasion assay, glycosylation and EMT characteristics following 24 h ICCM or CCCM-derived exosome incubation. (**A**) The data were presented as a mean of a total number of invaded cells, and the percentage of HPA and EMT markers (vimentin and E-cadherin) positive cells. The error bars represent the SEM of invasive cells and the percentage of HPA and EMT markers of 3 independent experiments. (*** *p* ≤ 0.0001). (**B**) Representative images for HPA labelling and EMT markers (vimentin and E-cadherin) assays in CCCM or ICCM-derived exosomes incubated MCF-7 cells. Scale bar: 100 μm.

In order to test if the previously observed bystander effects regarding invasive potential in MCF-7 cells are driven by exosomes; CCCM and ICCM-derived exosomes were transferred onto MCF-7 cells. A significant increase in the number of MCF-7 cells that invaded through Matrigel transmembrane system after 24 h of incubation with ICCM-derived exosomes compared to the MCF-7 cells incubated with CCCM-derived exosomes (Figure 3). HPA labelling showed a significant increase in the percentage of HPA positive MCF-7 cells after treatment with ICCM-derived exosomes compared to MCF-7 cells incubated with CCCM-derived exosomes, as also shown in Figure 3. MCF-7 cells incubated with ICCM-derived exosomes also showed a significantly increased vimentin positivity and reduced E-cadherin positivity showing an increased capacity to shift to a more EMT-like phenotype.

**Figure 4:**
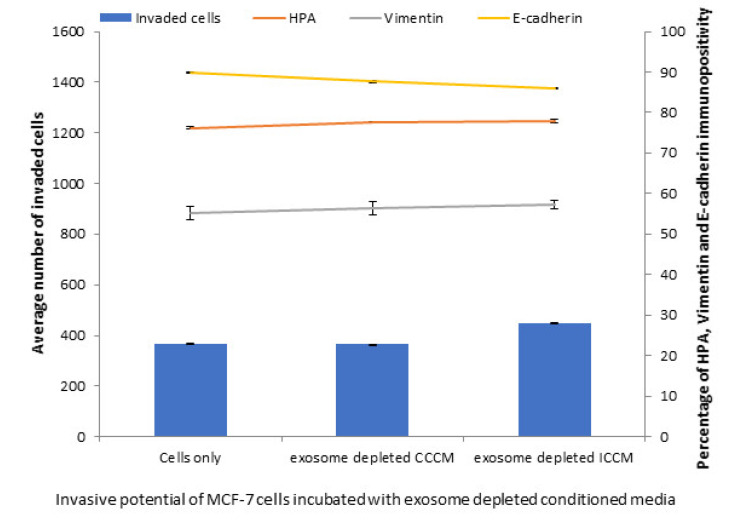
Invasive potential of MCF-7 cells shown by Matrigel invasion assay, glycosylation and EMT characteristics following 24 h incubation with exosome depleted ICCM or CCCM, as well as untreated control cells. The data were presented as a mean of a total number of invaded cells, and the percentage of HPA and EMT markers (vimentin and E-cadherin) positive cells. The error bars represent the SEM of invasive cells and the percentage of HPA and EMT markers of 3 independent experiments.

To further confirm that the exosomes are the responsible factors for the observed changes in MCF-7 cells, we analysed the number of invaded cells, HPA labelling, vimentin and E-cadherin immunopositivity in MCF-7 cells following 24 h incubation with exosome depleted CCCM and ICCM. The results showed contrary observations to ICCM exosome incubated cells; as exosome depleted ICCM caused no significant increase in the number of invaded cells, HPA labelling or a change in vimentin and E-cadherin immunopositivity of MCF-7 cells, compared to the cells incubated with exosome depleted CCCM (Figure 4).

#### 2.2.4. Vimentin and E-Cadherin Expression Levels

**Figure 5:**
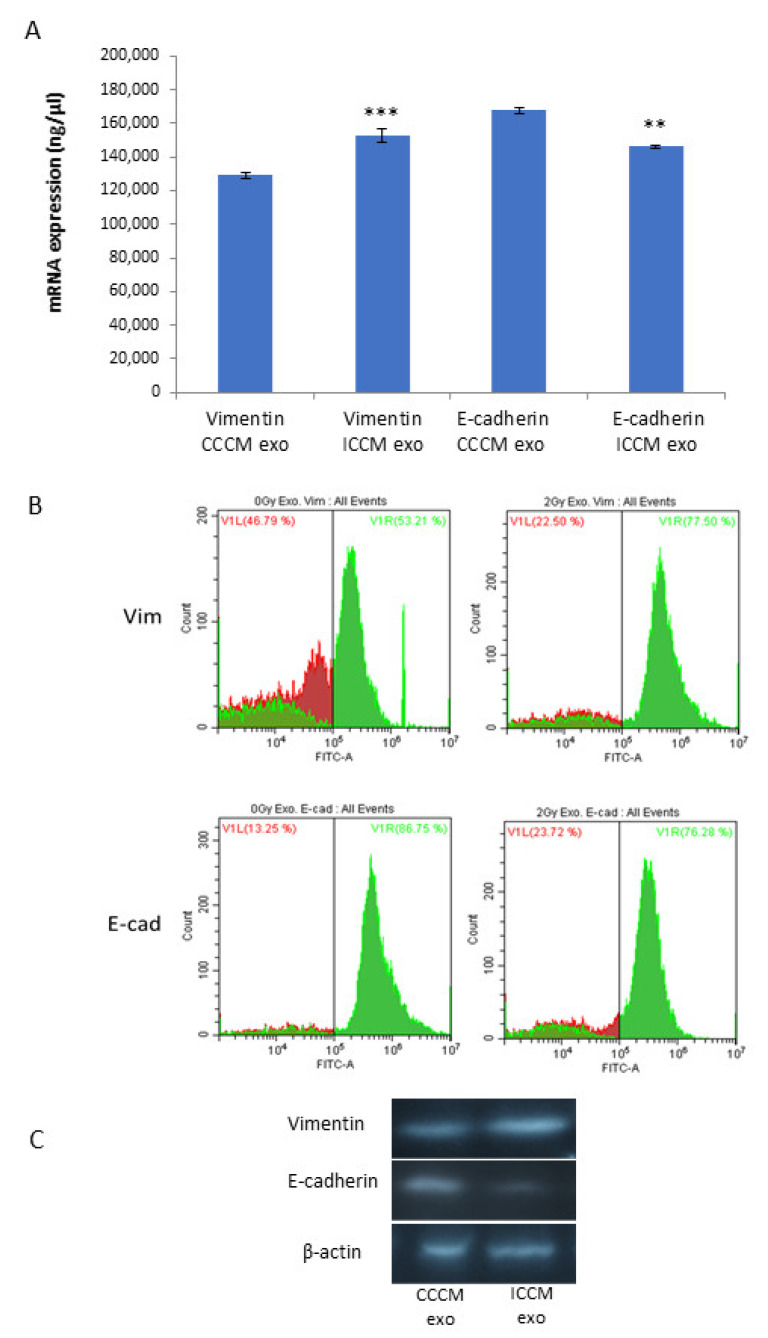
Comparison of vimentin and E-cadherin expression of MCF-7 cells incubated either with CCCM or ICCM-derived exosomes at the mRNA and protein levels. (**A**) qPCR analysis showing *vimentin* and *E-cadherin* expression in MCF-7 cells (** *p* ≤ 0.001, *** *p* ≤ 0.0001). The error bars represent SEM of the mRNA expression. The experiment was carried out in triplicate. (**B**) Flow cytometry analysis of MCF-7 cells. Data represent three independent experiments. (**C**) Representative Western blot analysis showing E-cadherin (97 kDa) and vimentin (54 kDa) expressions in MCF-7 cells.

Next, we aimed to investigate if changes that we observed with EMT marker assay following exosome incubation can be confirmed at the mRNA level. For this, we carried out qPCR analyses from the total RNA lysates of MCF-7 cells, following 24 h CCCM or ICCM-derived exosome incubation. Data demonstrated that mRNA transcript levels were significantly increased for *vimentin* and significantly reduced for *E-cadherin* in ICCM-derived exosome incubated cells compared to CCCM-derived exosomes incubated MCF-7 cells (Figure 5A). We also aimed to further confirm our findings via flow cytometry and Western blot analysis as shown in Figure 5B,C.

#### 2.2.5. qPCR Analysis of EMT Coupled Transcription Factors

**Figure 6:**
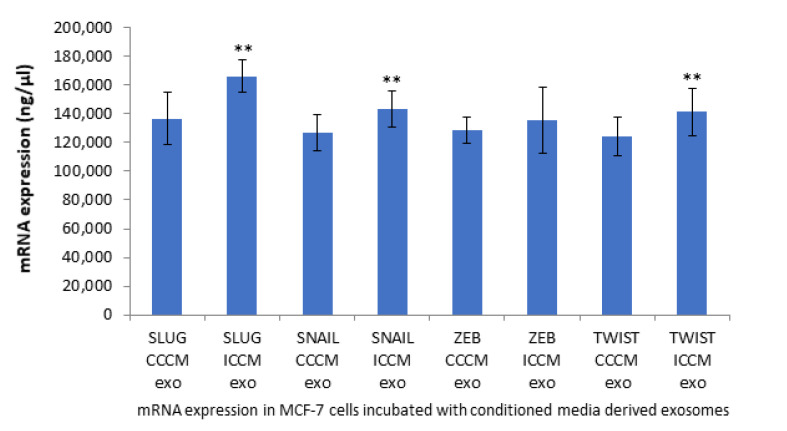
qPCR analysis showing expression of EMT coupled transcription factors *SLUG*, *SNAIL*, *ZEB* and *TWIST* in MCF-7 cells incubated either with CCCM or ICCM-derived exosomes. The error bars represent SEM of the mRNA expression (** *p* ≤ 0.001). The experiment was carried out in triplicate.

To further elaborate our study in the context of EMT like phenotype that was observed; the expression of EMT coupled transcription factors *SNAIL*, *SLUG*, *ZEB* and *TWIST* were investigated at the mRNA level. qPCR analysis showed all transcription factors with the exception of *ZEB* were significantly increased following incubation of MCF-7 cells with ICCM-derived exosomes compared to their expressions in MCF-7 cells incubated with CCCM-derived exosomes, as shown in Figure 6.

#### 2.2.6. TGF-β Expression Levels

**Figure 7:**
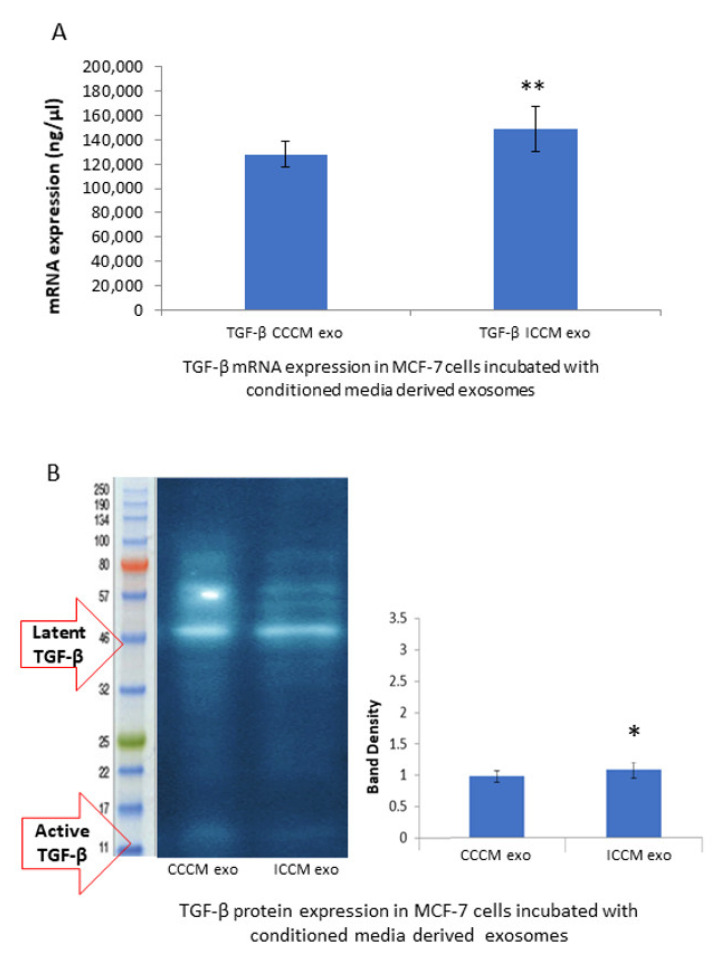
Comparison of TGF-β expressions at the mRNA and protein levels in the MCF-7 cells incubated either with CCCM or ICCM exosomes. (**A**) qPCR analysis showing *TGF-β* expression in MCF-7 cells. The error bars represent SEM of the mRNA expression. Experiment was carried out in triplicate. (**B**) Representative Western blot analysis image shows latent (44 kDa) and active (12.5 kDa) TGF-β expressions in MCF-7 cells. Bar diagram shows the TGF-β protein expressions measured as band density. The error bars represent the SEM of protein expressions of three independent experiments (* *p* ≤ 0.05, ** *p* ≤ 0.001).

Observation of a consistent increase in EMT markers and EMT promoting transcription factors in MCF-7 cells incubated with ICCM-derived exosomes, led us to check if this increase, may be, at least partially, derived by the master regulator of EMT, transcription factor TGF-β. qPCR analysis and the Western blot analysis showed that the TGF-β expression was increased at the mRNA (Figure 7A) and protein levels (Figure 7B) in the MCF-7 cells incubated with ICCM-derived exosomes compared to those incubated with CCCM-derived exosomes.

#### 2.2.7. GalNAc-T6 Expression

**Figure 8:**
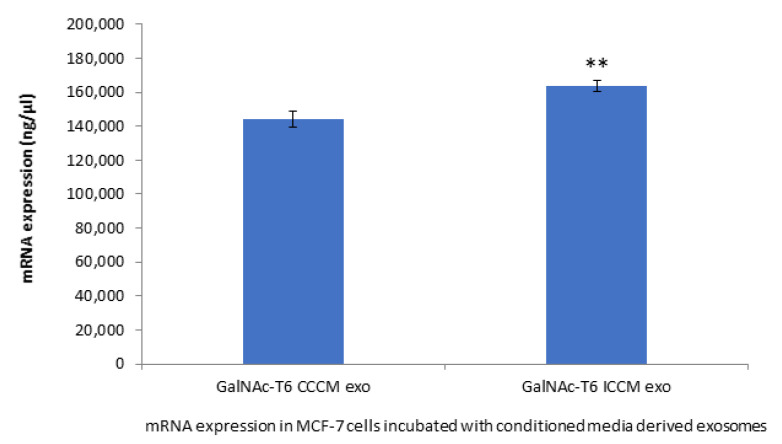
qPCR analysis showing expression of *GalNAc-T6* in MCF-7 cells incubated either with CCCM or ICCM-derived exosomes. The experiment was carried out in triplicate. The error bars represent SEM of the mRNA expression (** *p* ≤ 0.001).

To further support our findings that is related to HPA labelling, we chose to investigate the expression of GalNAc-T6 enzyme, which is the enzyme responsible for addition of first GalNAc residue to the polypeptide during glycosylation and, therefore, an increase in its expression might explain increased HPA labelling. Moreover, GalNAc-T6 enzyme is also linked to EMT process. The results depicted in Figure 8 shows that *GalNAc-T6* expression was increased significantly in MCF-7 cells incubated with ICCM-derived exosomes compared to those incubated with CCCM-derived exosomes.

### 2.3. Investigation of Exosome Cargo

In order to find the possible mechanism for the exosome bystander effects observed in the number invaded cells through Matrigel, EMT markers immunopositivity and glycosylation of MCF-7 cells, we aimed to investigate the changes in some potentially related miRNA cargo of exosomes from both CCCM and ICCM.

#### 2.3.1. Expression of *Let-7a*, *miR-30a*, *miR-200b*, *miR-9a* in Exosomes

**Figure 9:**
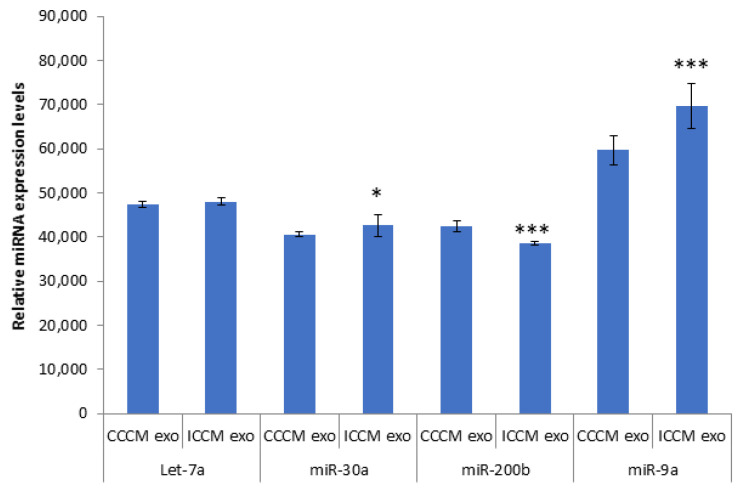
qPCR analysis showing expression of *Let-7a*, *miR-30a*, *miR-200b*, *miR-9a* in CCCM or ICCM-derived exosomes. Experiment was carried out in triplicate. The error bars represent SEM of the miRNA expression (* *p* ≤ 0.05, *** *p* ≤ 0.0001).

qPCR analysis was carried out for *Let-7a*, *miR-30a*, *miR-200b*, *miR-9a*. Data in Figure 9 demonstrated that there was a significant upregulation of *miR-30a* and *miR-9a* accompanied by a significant downregulation of miR-200b in ICCM-derived exosomes compared to CCCM-derived exosomes. No significant difference was observed for *Let-7a*.

#### 2.3.2. Expression of TGF-β Protein in Exosomes

**Figure 10:**
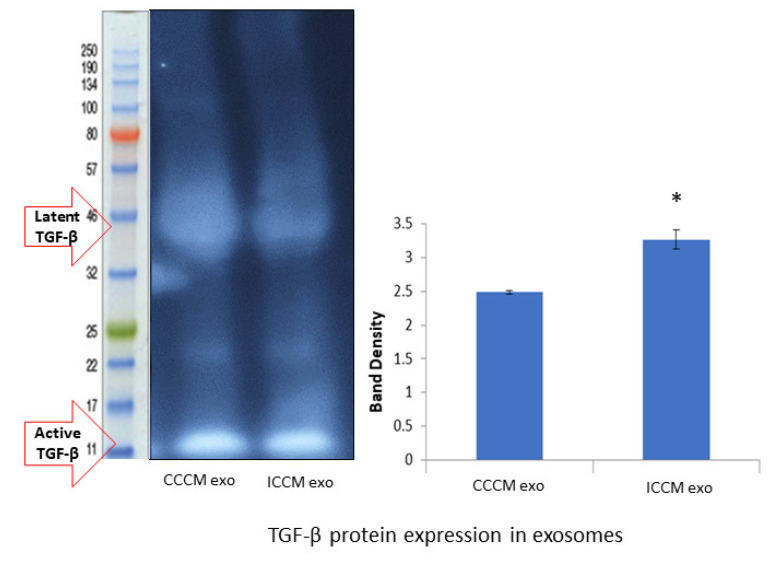
TGF-β protein expressions in the CCCM and ICCM-derived exosomes shown by Western blot analysis. Western blot image shows expected protein size for latent (44 kDa) and active (12.5 kDa) TGF-β. Bar diagram shows TGF-β protein expressions measured as band density. The error bars represent the SEM of protein expressions of three independent experiments (* *p* ≤ 0.05).

Finally, we investigated if TGF-β is differentially expressed in exosomes as another possible mechanism of increasing TGF-β and its downstream effectors causing exosome bystander effects in MCF-7 cells incubated with ICCM-derived exosomes. Western blot analysis showed increased TGF-β protein expression (Figure 10) in ICCM-derived exosomes compared to CCCM-derived exosomes.

#### 2.3.3. Exosome Cargo Inhibition Experiments

**Figure 11:**
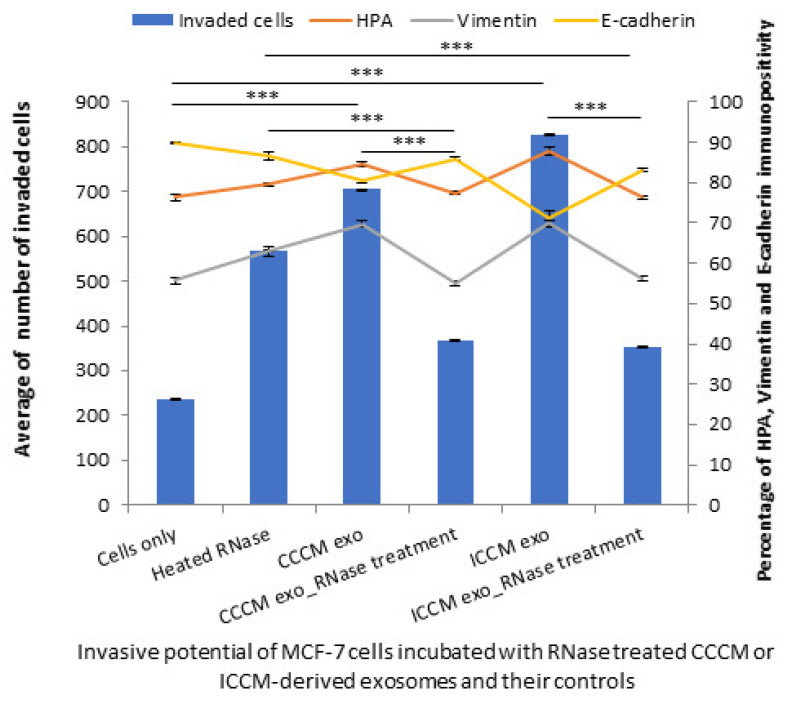
Invasive potential of MCF-7 cells shown by Matrigel invasion assay, glycosylation and EMT characteristics following 24 h heated RNase, CCCM-derived exosome, RNase-treated CCCM-derived exosome, ICCM-derived exosome and ICCM-derived RNase-treated exosome incubations. The data were presented as a mean of a total number of invaded cells, and the percentage of HPA and EMT markers (vimentin and E-cadherin) positive cells. The error bars represent the SEM of invasive cells and the percentage of HPA and EMT markers of 3 independent experiments. (*** *p* ≤ 0.0001).

In order to further confirm RNA and protein components from the exosomes are the major derivers of the changes observed in the invasive potential of MCF-7 cells; a series of experiments were designed where RNA or protein or both components of exosome cargo were inhibited. The results showed that the number of invaded MCF-7 cells were significantly reduced following incubations with RNase-treated CCCM-derived and ICCM-derived exosomes compared to the incubations with untreated CCCM-derived and ICCM-derived exosomes. Similarly, the percentage of HPA-positive and vimentin-positive cells were decreased while E-cadherin-positive cells were increased following incubation with RNase-treated CCCM-derived and ICCM-derived exosomes compared to their corresponding controls as demonstrated in Figure 11.

**Figure 12:**
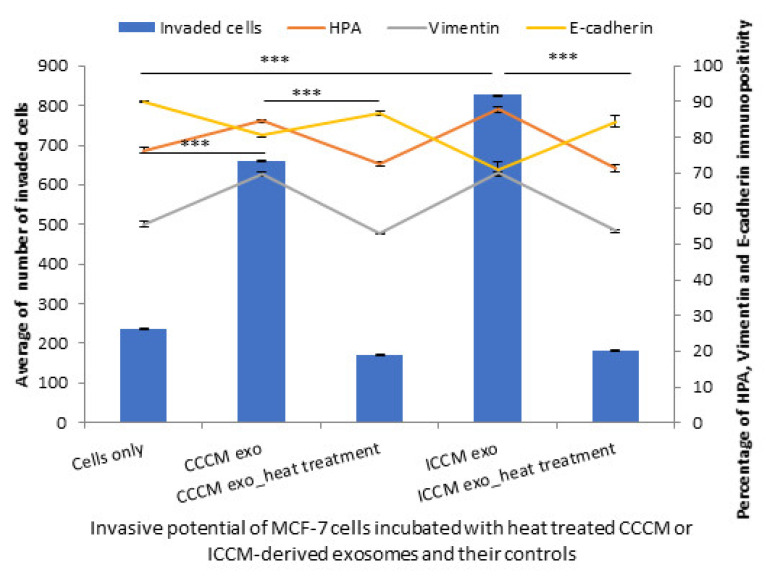
Invasive potential of MCF-7 cells shown by Matrigel invasion assay, glycosylation and EMT characteristics following 24 h post CCCM-derived exosome, heat-treated CCCM-derived exosome, ICCM-derived exosome and ICCM-derived heat-treated exosome incubations. The data were presented as a mean of a total number of invaded cells, and the percentage of HPA and EMT markers (vimentin and E-cadherin) positive cells. The error bars represent the SEM of invasive cells and the percentage of HPA and EMT markers of 3 independent experiments. (*** *p* ≤ 0.0001).

In parallel with these findings, a significant reduction was observed in MCF-7 cell invasiveness when they were incubated with heat-treated exosomes derived from CCCM and ICCM compared to those incubated with untreated CCCM-derived and ICCM-derived exosomes. Moreover, MCF-7 cells incubated with heat-treated exosomes also showed a significant decrease in percentage of HPA and vimentin-positive cells accompanied by an increase in E-cadherin-positive cells as shown in Figure 12.

**Figure 13:**
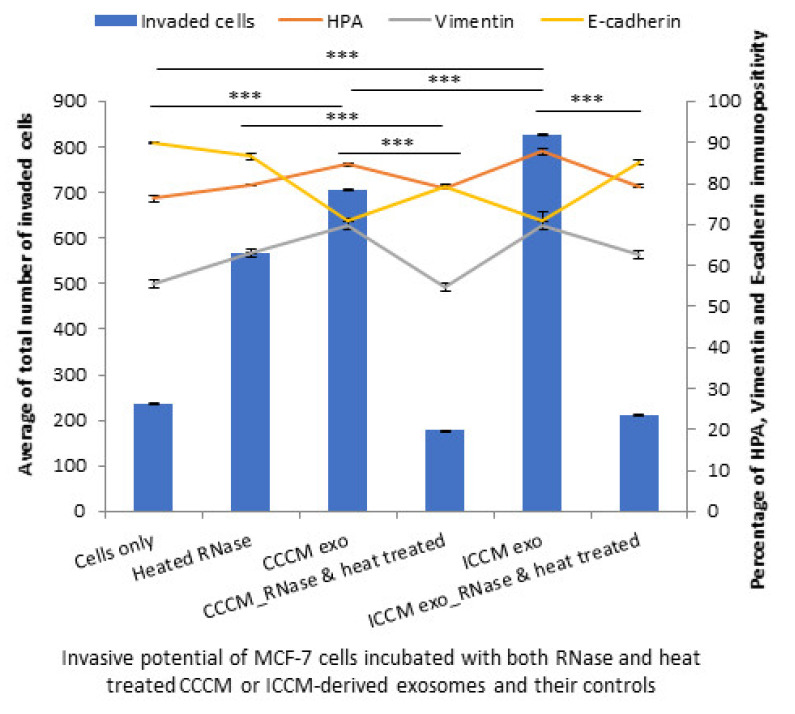
Invasive potential of MCF-7 cells shown by Matrigel invasion assay, glycosylation and EMT characteristics following 24 h post heated RNase, CCCM-derived exosome, RNase and heat treated CCCM-derived exosome, ICCM-derived exosome and ICCM-derived RNase and heat-treated exosome incubations. The data were presented as a mean of a total number of invaded cells, and the percentage of HPA and EMT markers (vimentin and E-cadherin) positive cells. The error bars represent the SEM of invasive cells and the percentage of HPA and EMT markers of 3 independent experiments. (*** *p* ≤ 0.0001).

Finally, inhibition of both RNA and protein cargo gave similar results to RNA or protein inhibition alone as the number of invaded MCF-7 cells were reduced following incubation with RNase and heat-treated exosomes compared to their corresponding controls. The percentage of HPA-positive and vimentin-positive cells were decreased, while E-cadherin-positive cells were increased following incubation with RNase and heat-treated CCCM-derived and ICCM-derived exosomes compared to those incubated with untreated CCCM-derived or ICCM-derived exosomes, as also shown in Figure 13.

## 3. Discussion

Breast cancer is the most frequently diagnosed cancer and the major cause of death among women worldwide [51]. Significant number of patients develop distant metastasis after initial therapeutic applications [52] including radiation therapy. Radiotherapy resistance and consequently tumor relapse and metastasis occur frequently in clinical settings, albeit the presence of advanced radiation therapy approaches [53].

Accumulating evidence shows that exosome-mediated signalling is one of the core mechanisms responsible for multidirectional communication of tumor cells and their associated microenvironment, which may result in enhancement of malignant tumor phenotypes and establishment of the pre-metastatic niche, collectively causing clinically detectable metastasis [54]. In parallel, recent studies have shown that exosomes and exosome-mediated signalling play a dynamic role in RIBE in cancer cell lines, many of which have focussed on the changes in miRNA and proteomic profiles of the exosomes or their effects on DNA damage [45,48,49,55,56,57]. There remains a lack of knowledge about how these alterations are reflected in other functional characteristics of cells from the aspects of invasiveness and metastasis. Hence, in the current study, we focused on exosome-mediated bystander effects (exosome bystander effects) of 2 Gy X-ray therapeutic level of ionizing radiation, in the context of in vitro invasive behaviour of MCF-7 breast cancer cells, while also investigating underlying plausible molecular mechanisms.

Results of the conditioned media transfer experiments provided us the first clues about the enhanced invasiveness of MCF-7 cells as MCF-7 cells incubated with ICCM showed higher number of invaded cells through Matrigel membrane system, HPA-positivity as well as EMT-like changes in terms of immunopositivity compared to MCF-7 cells incubated with CCCM as shown in Figure 1.

Characterisation of ICCM and CCCM-derived exosomes demonstrated that both exosome concentration and exosome diameter were increased compared to exosomes isolated from unirradiated MCF-7 cells (CCCM exosomes) (Figure 2A), which agree with previous findings in the literature [49]. Incubation of MCF-7 cells with ICCM or CCCM-derived exosomes demonstrated that the number of invaded cells, HPA-positive cells, vimentin-positive cells were increased, while the number of E-cadherin-positive cells were decreased, as an indicator of a shift to a more EMT-like and invasive phenotype in ICCM-derived exosomes incubated MCF-7 cells (Figure 3). Findings above were further solidified by the observation that exosome depleted ICCM or CCCM medium incubated MCF-7 cells did not show a significant difference in terms of the number of invaded cells, HPA-positivity or EMT marker immunopositivity, as shown in Figure 4. These findings collectively suggest that that the bystander effects observed regarding the invasive behaviour of MCF-7 cells incubated with ICCM-derived exosomes, can be solely attributed to the presence of exosomes and the content of the exosome cargo.

EMT is often considered to be the primary driving force of the metastatic cascade and, thus, EMT related changes have been explored in this study. The loss of key intercellular adhesion protein E-cadherin is regarded as a hallmark of EMT which in turn contributes to metastasis by enhancing the ability of cells to migrate and invade [58]. On the contrary, vimentin, an intermediate filament protein characteristically upregulated in cells undergoing EMT, plays a key role in the motility and migration of breast cancer cells as it is also highly expressed at the wound edge in mammary epithelial cells and breast cancer cells [59,60]. Studies have also demonstrated that knockdown of vimentin resulted in downregulation of genes involved in breast cancer invasion and the basal-like phenotype, including Axl, ITGB4, and PLAU, with a subsequent upregulation in the genes abundant in normal mammary epithelium, including RAB25 and EHF [61].

Results of qPCR analysis (Figure 5A) showed increased *vimentin* and reduced *E-cadherin* expressions in ICCM-derived exosome-incubated cells compared to CCCM-derived exosome-incubated controls, while the same expression trend was also observed by Flow cytometry (Figure 5B) and Western blot analysis (Figure 5C). These findings not only consolidate our findings regarding EMT-like changes in immunopositivity of MCF-7 cells incubated with ICCM-derived exosomes, but also indicates that the expressions of these proteins were deregulated at the transcription level.

The loss of EMT epithelial gene expression and activation of a mesenchymal molecular profile can involve the core set of EMT-coupled transcription factors, SNAIL, SLUG, TWIST, ZEB1 [62]. SNAIL and SLUG have long been characterized as strong repressors of E-cadherin expression. It has been shown that in MDA-MB-231 breast cancer cells, silencing of SNAIL increases E-cadherin expression and, consequently, decreases expression of mesenchymal markers, and inhibits their invasiveness [63]. Similarly, studies in MCF-7 and Hs578T breast cancer cells have shown that TWIST binds to the E-cadherin promoter and it down regulates E-cadherin promoter activity as shown by chromatin immunoprecipitation assays and promoter reporter assays respectively [64]. Furthermore, Twist expression was found to be inversely correlated with E-cadherin expression in clinical breast cancer samples. Moreover, ZEB1 represses EB1 transcription of E-cadherin by binding to two E-box sequences in its promoter region [65,66].

As demonstrated in Figure 6, qPCR analysis of EMT coupled transcription factors *SLUG*, *SNAIL* and *TWIST* in ICCM-derived exosome-incubated cells showed increased upregulation of these transcription factors (TFs), whilst no change was observed in *ZEB1* expression compared to CCCM-derived exosome-incubated cells. Upregulated *SLUG*, *SNAIL*, and *TWIST* may at least partially explain the EMT-like phenotype that we have observed with ICCM-derived exosome-incubated MCF-7 cells, i.e., through the repression of E-cadherin expression. ZEB1 has previously been shown to function in the lineage differentiation from multipotent mesenchymal stem cells as well as being linked to the regulation of cellular metabolism [67,68], which distinguishes it from other EMT coupled TFs and this difference could be a possible explanation for unchanged *ZEB1* levels reported here.

Moreover, the expression of EMT promoting cytokine TGF-β was found to be upregulated at mRNA (Figure 7A) and protein (Figure 7B) levels in MCF-7 cells incubated with ICCM-derived exosomes compared to the control cells. TGF-β has a pivotal role in EMT regulation and metastasis [22,23,24] and the consistent increase that we have observed in EMT-like immunopositivity, EMT-promoting markers and TFs may be partially attributed to this upregulation.

GalNAc-T6 enzyme catalyses the initial reaction in O-linked oligosaccharide biosynthesis with the transfer of N-acetyl-D-galactosamine residue to a serine or threonine on the protein receptor [69], which can be accounted for increased HPA positivity in cells. Aberrant expression of the enzyme has been linked with oncogenic transformation as well as metastasis [70,71,72]. In parallel with the increased percentage of HPA-positive MCF-7 cells upon incubation with ICCM-derived exosomes (Figure 3), we found that GalNAc-T6 enzyme expression was increased in ICCM-derived exosome-incubated MCF-7 cells compared to control cells incubated with CCCM-derived exosomes as shown in Figure 8. GalNAc-T6 enzyme is not only important for its functions in glycosylation but also as a promoter of EMT. Recent data indicate that overexpression of the enzyme caused distortion of acinar morphogenesis resulting in cellular changes similar to those of EMT in normal mammary epithelial MCF10A cells. In addition, GalNAc-T6 stabilised O-glycosylated fibronectin (FN), which further enhanced the acinar distortion [73]. Moreover, it has been shown that oncofetal FN (onfFN), which requires O-glycan addition at a specific Thr, GalNAc-T3, and/or GalNAc-T6, was up-regulated in TGFβ-induced EMT, whereas inhibition of GalNAc-T3 and GalNAc-T6 suppressed the EMT process [74]. Moreover, in a follow-up study, only O-glycosylated onfFN was shown to induce EMT-related events [75]. Therefore, increased GalNAc-T6 activity may be also contributing to enhanced EMT-like phenotype in ICCM-derived exosome incubated cells.

Due to the observation of consistent change in the number of invading cells through Matrigel, EMT marker immunopositivity and glycosylation in ICCM-derived exosome-incubated cells, we decided to elaborate on our findings by investigating exosome cargo. It is evident in literature that crosstalk between miRNAs and EMT-TFs is also critical for EMT regulation [76]. Moreover, recent studies have shown that miRNAs play crucial roles in radiation response and intercellular gene-based communication between irradiated and bystander cells [48,77,78,79]. Exosomes released from cancer cells are also shown to promote tumorigenesis through performing cell-independent microRNA biogenesis [80]. Therefore, we were motivated to investigate some miRNAs in exosomes that could be potentially deregulated and be responsible for the functional and expressional changes we have observed. As also depicted in Figure 9, the qPCR analysis showed an upregulation of *miR-30a* and *miR-9a* accompanied by a significant downregulation of *miR-200b* in ICCM-derived exosomes compared to CCCM-derived exosomes while there was no significant difference for *Let-7*. miR-9 has been reported to promote EMT and metastasis in breast cancer cells, through suppression of E-cadherin [81]. On the other hand, miR-200 has been repeatedly shown to have a crucial role in EMT, as the expression of miR-200 family members were found to be highly associated with the epithelial phenotype of cancer cells [82,83,84].

Despite its secretion as soluble homodimers by carcinomas; TGF-β is also secreted in association with exosomes which can trigger both canonical and non-canonical signalling pathways [85,86]. Moreover, it has been shown that TGF-β is relevant to medium mediated RIBE [87]. Our primary observation regarding TGF-β protein in exosomes is that its expression is increased in ICCM-derived exosomes compared to CCCM-derived exosomes, suggesting that TGF-β is directly transferred and causing downstream effects in recipient bystander cells. Interestingly, we observed that the active form of TGF-β was higher than the latent form of the protein in exosomes (Figure 10), while the latter was found to be the dominant form in the cellular lysate (Figure 7B). These results further suggest that TGF-β required for inducing EMT-like changes may be mainly being sourced from exosomes uptaken in ICCM-derived exosome-incubated MCF-7 cells.

Importantly, RNA (Figure 11) and protein inhibition (Figure 12) experiments suggest that both proteins, particularly active TGF-β, and specific miRNA cargo in ICCM-derived exosomes may have significant roles in increased metastatic behaviour observed in bystander cells and both are required to induce those changes (Figure 13).

Overall, in this study, the data shows that exosomes secreted from irradiated cells render bystander cells more invasive, there is more O-linked GalNAc glycosylation and an EMT-like phenotype, which may also be provoking one other, and collectively enhance the invasiveness metastatic activity of exosome bystander cells. Moreover, this modulation could be triggered by an increase in active TGF-β along with regulatory miRNA-9, at least to some extent.

**Figure 14:**
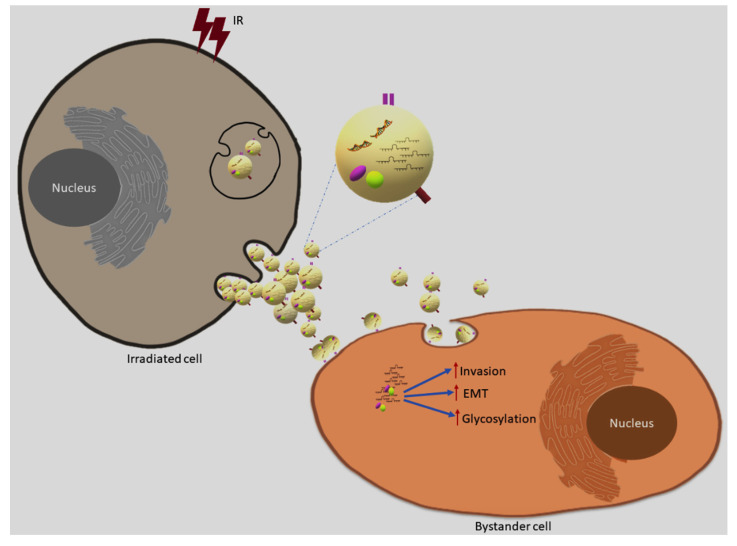
Exosome mediated bystander effects. Ionising radiation can modulate exosome cargo and promote invasion, EMT and glycosylation enhancing invasive phenotype in the bystander cells via uptake of exosomes released from directly irradiated cells.

In conclusion, as outlined in Figure 14, here we demonstrate for the first time that ionising radiation can modulate exosome cargo to induce an invasive phenotype in the context of RIBE, providing evidence that exosomes can promote radiation resistance through enhanced invasive phenotype. We believe that these findings may have implications to develop new strategies in cancer radiotherapy and overcome radioresistance.

## 4. Materials and Methods

### 4.1. Cell Culture

Breast epithelial cancer cell line MCF-7 cells were kindly provided by Joestein Dahle (Institute for Cancer Research, Oslo, Norway). They were cultured in T75 or T175 flasks, or on coverslips in 12-well plates, in Dubelcco’s Modified Eagle’s Medium/Nutrient Mixture F-12 Ham media (D6421, Sigma, St. Louis, MO, USA) supplemented with 13% inactivated fetal bovine serum (FBS) (F7524, Sigma, St. Louis, MO, USA); 2 mM L-glutamine (25030149, Gibco, Invitrogen, Paisley, UK) and 1% (*v*/*v*) penicillin/streptomycin solution (P0781, Sigma, St. Louis, MO, USA) in a humidified 5% CO_2_ incubator at 37 °C.

### 4.2. Irradiation

MCF-7 cells at 70% confluency were irradiated with therapeutic dose of 2 Gy X-ray using MXR321 X-ray machine at 250 kV constant potential, 14 mA, and a dose rate of 0.53 Gy/minute for 3.56 min. All irradiation experiments were conducted at the Gray institution, University of Oxford, Oxford, UK.

### 4.3. Conditioned Media Transfer

MCF-7 cells subcultured in T75 tissue culture flasks for four generations were either irradiated at 70% at 2 Gy X-rays or sham-irradiated. At 4 h post-irradiation, ICCM and CCCM were transferred to a flask of unirradiated cells at 70% cell confluence. Following 24 h of incubation at 37 °C, 5% CO_2_, cells were seeded onto coverslips in 12-well plates for glycosylation and EMT markers assays.

### 4.4. Exosome Isolation, Purification and Characterisation

MCF-7 cells were subcultured in T175 (seeding density is 3.0 × 10^6^ cells in 25 mL media) tissue culture flasks for four generations were either irradiated at 70% at 2 Gy X-rays or sham-irradiated. At 4 h post irradiation, CCCM and ICCM were collected in 50 mL sterile conical polypropylene centrifuge tubes and subjected to centrifugation at 2000× *g* for 15 min. The supernatants were transferred to fresh tubes and subjected to centrifugation at 14,000× *g* for 30 min. Finally, exosomes were pelleted by centrifugation at 120,000× *g* for 90 min in 40 mL cellulose propionate tubes (344058, Beckman Coulter, Brea, CA, USA) and pellets were resuspended in 500 µL of PBS. Exosome size and concentration were determined via qNano machine and software by using NP100 nm nanopore by the method previously described in detail in [44,45].

### 4.5. Exosome Incubations

For exosome incubations, 50 μL (~2.5 × 10^9^) or 500 μL (~2.5 × 10^10^) of exosomes isolated from 2 Gy X-ray-irradiated or sham-irradiated cells were transferred onto MCF-7 cells cultured on coverslips in 12-well plates or T75 tissue culture flasks, respectively. Cells were analysed for invasion through Matrigel, glycosylation and EMT markers, flow cytometry, qPCR and Western blot analysis following incubation with exosomes for 24 h at 37 °C, 5% CO_2_.

### 4.6. Inhibition of Exosome Cargo (RNase-A and Heat Treatments)

Exosome cargo was released by collecting exosome pellets into distilled water. Subsequently, inhibition of exosome cargo was achieved as described previously [45,88] by either incubation of ~2.5 × 10^9^ or ~2.5 × 10^10^ exosomes at 37 °C for 30 min in the presence of 30 µg/mL RNase-A (10109142001, Sigma, St. Louis, MO, USA) or incubation of exosomes at 100 °C in a water bath for 10 min, or by carrying out both steps sequentially.

### 4.7. Invasion Assays

A Matrigel invasion assay is one of the key functional assays for evaluation of EMT and invasive capacity of cancer cells [89]. Briefly, cells were seeded onto membranes coated with Matrigel (0.5 mg/mL). Following 24 h incubation at 37 °C, cell media was replaced by ~2.5 × 10^9^ of ICCM-derived exosomes, CCMM-derived exosomes or inhibited exosome cargo in serum free media, or ICCM and CCMM, respectively. After 24 h, media and the Matrigel were discarded. Inserts were washed twice with PBS and fixed with 3:1 methanol-acetic acid solution. Finally, membranes were stained with haematoxylin and eosin and mounted on microscope slides. Three slides were prepared for each sample, and 10 images were taken per slide. Total number of invaded cells were scored using Axioplan Light Microscope (Zeiss, Germany) with colour camera (ProgRes, Germany), under 200× magnification.

### 4.8. Immuno- and Lectin Cytochemistry

MCF-7 cells grown on coverslips in 12-well plates and cell media were replaced by (~2.5 × 10^9^) of ICCM-derived exosomes, CCMM-derived exosomes or inhibited exosome cargo in serum free media, or ICCM and CCMM. Following 24 h of incubation, cells were fixed 4% formaldehyde in PIPES buffer (P1851, Sigma, St. Louis, MO, USA) at 4 °C for 15 min. Following a wash step with PIPES buffer, cells were permeabilised with 0.1% *v*/*v* Triton X100 (T9284, Sigma, St. Louis, MO, USA). After three washes with PIPES buffer, endogenous peroxidase was blocked with 3% *v*/*v* methanol/hydrogen peroxide for 20 min at room temperature (RT). The cells were washed three times with 1X Tris buffered saline (TBS) (T5030, Sigma, St. Louis, MO, USA) and blocked with 3% *w*/*v* bovine serum albumin (BSA) in TBS for 30 min. For EMT markers, vimentin and E-cadherin, cells were incubated with either 2 µg/mL of rabbit polyclonal anti-vimentin antibody (ab137321, Abcam, Cambridge, UK) or 0.5 µg/mL of rabbit monoclonal anti-E-cadherin antibody (ab76319, Abcam, Cambridge, UK) at 4 °C overnight. Cells were washed three times with PBS and then incubated with 4 μg/mL donkey anti-rabbit IgG H&L horseradish peroxidase (HRP) (ab6802, Abcam, Cambridge, UK) for 1 h. For detection of GalNAc glycosylation, cells were incubated with 10 μg/mL biotinylated HPA [90] (L6512, Sigma, St. Louis, MO, USA) in 1% BSA/TBS for 3 min and then with 5 µg/mL avidin peroxidase (A3151, Sigma, St. Louis, MO, USA) in 1% BSA/TBS for 30 min. In all cases this was followed by incubation with DAB peroxidase substrate (SK-4100, Vector Laboratories, Burlingame, CA, USA) and cells were counterstained with haematoxylin, dehydrated and mounted. They were visualised using Axioplan Light Microscope (Zeiss, Germany) with colour camera (ProgRes, Germany), under 400× magnification. Three slides were prepared for each sample, and 10 images were taken per slide. Immuno-positive cells were counted using Image J software and data was presented as the percentage of labelled cells to a total number of cells.

### 4.9. Flow Cytometry

For the flow cytometry [91], MCF-7 cells were grown and incubated with either ~2.5 × 10^10^ ICCM or CCCM-derived exosomes in T75 flasks for 24 h and then collected into 50 mL centrifuge tubes, washed three times with PBS and fixed with 80% methanol for 5 min and further washed three times with PBS. The cells were then permeabilised with 1% Triton X-100 in PBS for 10 min. Following three washing steps with PBS, cells were incubated with 2 µg/mL rabbit polyclonal anti-vimentin antibody (ab137321, Abcam, Cambridge, UK) and rabbit monoclonal anti-E-cadherin (ab76319, Abcam, Cambridge, UK) primary antibodies for 1 h at RT. Then cells were washed three times with TBS-T buffer and then incubated with 2 μg/mL AlexaFluor^®^ 488-conjugated polyclonal goat anti-rabbit IgG (ab150077, Abcam, Cambridge, UK) for 30 min at RT in the dark. Cell suspensions were analysed using flow cytometer (Cytoflex 5, Beckman Coulter, Brea, CA, USA). An FITC-A filter was applied, and data were presented as a histogram of positive cells to vimentin and E-cadherin via use of CytExpert 2.1 software.

### 4.10. Reverse Transcription and Quantitative Polymerase Chain Reaction

Total RNA from 2 × 10^6^ MCF-7 cells or exosomal RNA, from media that was collected from 20–24 × 10^6^ cells, were isolated using RNeasy^®^ Mini kit (74104, Qiagen, Hilden, Germany) according to manufacturer’s instructions. OD260/280 nm absorbance ratios were between 1.7 to 2.1 for all RNA isolations. Isolated RNA was treated with DNase-1 (AMPD-1, Sigma, St. Louis, MO, USA) and converted to cDNA by use of iScript™ cDNA synthesis kit (1708891, Bio-Rad, Hercules, CA, USA) according to the manufacturer’s instructions. Gene amplification was achieved by iTag universal SYBER Green Supermix (172-5122, Bio-Rad, Hercules, CA, USA). Briefly, 10 µL iTaq universal SYBER green Supermix was mixed with 1 µL of cDNA (500 ng/µL), 2 µL of forward and reverse primers (Table 1) and 7 µL of nuclease-free water. Three replicates were analysed for each sample. The reaction started at 95 °C for 2 min and followed by 39 cycles of 95 °C for 10 s and 60 °C for 30 s in Thermal Cycler (Bio-Rad, Hercules, CA, USA). Cq data were displayed/analysed using the qPCR software.

### 4.11. Western Blot

Western blotting was used as semi-quantitative technique in order to compare the protein expression levels in the cells [92] and exosomes [44]. Proteins were isolated from 2 Gy X-ray-irradiated and sham-irradiated MCF-7 cells, MCF-7 cells treated with exosomes from irradiated MCF-7 cells or unirradiated MCF-7 cells, or directly from exosomes isolated from irradiated and non-irradiated MCF-7 cells using RIPA Lysis Buffer (20-188, Millipore) in the presence of Protease Inhibitor Cocktail (P8340, Sigma, St. Louis, MO, USA). Total protein was measured using the Bradford assay by use of Coomassie blue solution and Pierce™ Bovine Serum Albumin Standard Pre-Diluted Set (23208, Thermo Scientific, Waltham, MA, USA).

20 µg of whole cell lysates or exosomal proteins were mixed with RunBlue LDS sample buffer (NXB31010, Expedeon; Abcam, Cambridge, UK) in the presence of DTT and separated in Mini-PROTEAN^®^ TGX Stain-Free™ Protein Gels (456-8126, Bio-Rad, Hercules, CA, USA) and transferred onto Amersham^TM^ Hybond^TM^ PVDF membrane (10600090, GE Healthcare, Little Chalfont, UK). The membranes were blocked in 5% BSA in TBS-T buffer and then incubated with anti-CD63 (ab134045, Abcam, Cambridge, UK), anti-TSG101 (ab125011, Abcam, Cambridge, UK), anti-TGFβ-1 (ab179695, Abcam, Cambridge, UK), rabbit polyclonal anti-vimentin (ab137321, Abcam, Cambridge, UK), rabbit monoclonal anti-E-cadherin antibody (ab76319, Abcam, Cambridge, UK) at 1:500 dilution, overnight. Membranes were washed three times with TBS-T buffer and then incubated with goat polyclonal antibody to rabbit IgG H&L (Alexa Fluor^®^ 488) (ab150077, Abcam, Cambridge, UK) for 1 h at 1:1000 dilution. Bands were visualised using Chemi™Doc MP Imaging system (Bio-Rad, Hercules, CA, USA) and analysed by Image Lab 4.1 software.

### 4.12. Statistical Analysis

Exosome size and diameter and viability data significance was tested by Student’s t-test using raw data. For HPA binding and EMT marker assays percentage of labelled cells to total cells were tested by Fisher’s exact test. Invasion assay data was also subjected to Fisher’s exact test, using the median of the raw data. qPCR and Western blot results were represented as mean ± SEM. Each experiment was carried out in triplicates. Data were considered statistically significant if *p*-value was lower than 0.05 (* *p* < 0.05, ** *p* < 0.01, *** *p* < 0.001).

## Figures and Tables

**Table 1:** Forward and reverse primer sequences used for qPCR detection.

Target Gene	Primer Sequence (5′-3′)	
*Vimentin*	F: ATGGCTCGTCACCTTCG R: AGTTTCGTTGATAACCTGTCC	Primers for EMT related genes
*E-cadherin*	F: ACGCATTGCCACATACA R: CGTTAGCCTCGTTCTCA	
*TGFβ-1*	F: TAAAGGGTCTAGGATGCGCG R: GACTTTTCCCCAGACCTCGG	
*SLUG*	F: AGCAGTTGCACTGTGATGCC R: ACACAGCAGCCAGATTCCTC	
*SNAIL*	F: AATCGGAAGCCTAACTACAGCG R: GTCCCAGATGAGCATTGGCA	
*ZEB1*	F: TCCCTGCCAAGAACAATGATCA R: AGGTGATGGGGATGGTGTACTA	
*TWIST*	F: ACAGCCGCAGAGACCTAAAC R: GGCCTGTCTCGCTTTCTCTT	
*GalNAc-T6*	F: AGAGACAGGGCAGAGGGTAG R: CCTTTGTCATGGCATCCCCT	Primers for glycosylation related gene
*has-Let -7a*	F: GGGGCTAATACTGCCTGGTAA R: TTCACAATGCGTTATCGGATGT	Primers for exosomal miRNA
*has-miR-200b*	F: GTTAGAATTAGGGTTTTTGGGGAGG R: ACCTATCAAACTTCTCAATATAAAC	
*has-miR-30a*	F: GGGATTCTGAAGGTGGGTGG R: AAGAGAGGCAGCTTTCACCC	
*has-miR-9f*	F: CCAAGCTTATAAGTGAGCGCATTC R: CGGAATTCGTGTTGGAGAACAGCA	

*GalNAc-T6*: N-acetylgalactosaminyltransferase-6 (also known as ppGalNAc-T6); *TGFβ-1*: transforming growth factor β1; F: forward; R: reverse.

## Data Availability

The data including the raw data are stored in the, Student logbook and hard external hard drive. This information will be stored in the department.
